# Experimental study on the regulation of erlotinib-induced radiosensitization with an anti-c-MET monoclonal antibody

**DOI:** 10.1186/s12935-014-0109-5

**Published:** 2014-11-30

**Authors:** Hong-Qing Zhuang, Hongxia Zhuang, Qifu Bo, Yihang Guo, Jun Wang, Lu-Jun Zhao, Zhi-Yong Yuan, Ping Wang

**Affiliations:** Department of Radiotherapy, Tianjin Medical University Cancer Institute and Hospital, National Clinical Research Center for Cancer, Tianjin Key Laboratory of Cancer Prevention and Therapy, Tianjin, China; Tianjin Lung Cancer Center, Tianjin, China; Department of Hematology, Weifang People’s Hospital, Weifang, Shandong province China; Department of Oncology, Affiliated Hospital of Weifang Medical University, Weifang, Shandong province China

**Keywords:** Tyrosine kinase inhibitor, Radiosensitization, Radiation resistance, Acquired drug resistance, PI3K pathway

## Abstract

**Purpose:**

Erlotinib is a novel therapeutic agent for cancer treatment. This study was performed to investigate the role of c-MET-PI3K-AKT pathway in the regulation of erlotinib-induced radiosensitization.

**Methods:**

A973 lung adenocarcinoma cells treated with 6 Gy of radiation were incubated in the presence of erlotinib. The apoptotic rate after 24 hours, the colony-formating rate after 14 days, and changes in the c-MET expression levels after 14 days of irradiation were examined. Surviving fractions in different treatment groups (blank control, radiation alone, erlotinib alone, anti-c-MET monoclonal antibody alone, combined erlotinib and radiation, and combined erlotinib and radiation with anti-c-MET monoclonal antibody groups) were determined, the survival curves were plotted, and the sensitizer enhancement ratio was calculated using colony formation assays. Expressions of c-MET, p-c-MET, PI3K, AKT, and p-AKT in cells in different treatment groups were examined by Western blot analysis.

**Results:**

The apoptotic rate in the combined erlotinib and radiation group was higher than those in single treatment groups; however, the colony-forming rate remained approximately 2.04 ± 1.02%. The expression of c-MET in colony-forming cells in the combined group significantly increased, and the blockade of c-MET activity significantly enhanced the radiosensitizing effect of erlotinib. The expression of c-Met, p-c-MET, PI3K, AKT, and p-AKT among colony-forming cells significantly decreased upon the inhibition of c-MET.

**Conclusions:**

Upregulated activity of the c-MET-PI3K-AKT pathway was found to be important for cell survival under combined the treatment with erlotinib and radiation. The blockade of the c-MET-PI3K-AKT signaling pathway enhanced the radiosensitizing effect of erlotinib.

## Introduction

A number of previous studies have demonstrated a clear radiosensitizing effect of erlotinib treatment [1-3]. However, the combined treatment with erlotinib and radiation sometimes exhibits poor antitumor effects [4]. Our previous report suggested that the extent of antitumor effects of the combined erlotinib and radiation treatment may be related to the PI3K pathway [5], although the underlying mechanism remains unclear. Therefore, the study of signaling pathways related to erlotinib-induced radiosensitization, understanding the survival mechanisms of tumor cells under the combined treatment with erlotinib and radiation, and further exploration of methods to enhance the radiosensitizing effect of erlotinib may have potential clinical significance.

## Materials and method

### Reagents and cell culture

RPMI-1640 culture medium was obtained from Gibco (USA, Grand Island), and fetal bovine serum was obtained from Hangzhou Sijiqing Biological Engineering Materials Co., Ltd. Monoclonal antibodies targeting c-MET, phosphorylated c-MET, PI3K, AKT, and phosphorylated AKT were purchased from Santa Cruz Biotechnology, Inc. (USA, Dallas, Texas). RNase A and propidium iodide (PI) were obtained from Sigma (USA, St. Louis, MO). The CO_2_ incubator used for cell culture was purchased from Heraeus (Germany, Frankfurt), and the high-speed refrigerated centrifuge was also obtained from Heraeus. The flow cytometer was from Beckman Coulter, Inc. (USA, California). The A973 lung adenocarcinoma cell line was used in this study. A973 cells expressed high levels of epidermal growth factor receptor (EGFR) and phospho-EGFR, as reported previously [5-7]. Cells were cultured in RPMI-1640 medium supplemented with 10% fetal bovine serum, 100 IU/ml penicillin, and 100 IU/ml streptomycin in a 37°C incubator with an atmosphere of 5% CO_2_. Cells in the exponential growth phase were irradiated.

### Colony formation assay

Colony-forming rates of the tumor cells were determined using the colony formation assay. The experiments on erlotinib-induced radiosensitization included the following treatment groups: blank control group, radiation alone group, erlotinib alone group, anti-c-MET monoclonal antibody alone group, combined erlotinib and radiation group, and combined erlotinib and radiation with anti-c-MET monoclonal antibody group. Cells in the exponential growth phase were trypsinized, counted, diluted, and seeded onto 35-ml flasks. The number of cells seeded onto the flasks was adjusted according to the radiation dose (500, 1000, 2000, 4000, 6000, 8000, and 10000 cells were seeded in 0, 1, 2, 4, 6, 8, and 10 Gy groups, respectively). The concentrations of erlotinib and anti-c-MET monoclonal antibody used were 20 nM. and 10 nM, respectively. A radiation dose of 2 Gy/min was selected, and cells were exposed to 0, 1, 2, 4, 6, 8, or 10 Gy of radiation after the attachment of cells on the plastic at 37°C, 5% CO_2_, and constant humidity. After 14 days of cell seeding, the culture dishes were collected, and the culture medium was discarded. Cells were fixed and subjected to Giemsa staining. The number of colonies containing more than 50 cells was counted, and the cell survival fraction (SF) was calculated. The experiments were confirmed three times, and each treatment group contained three parallel samples. The single-hit, multitarget model was used to fit the cell survival curves [8-10].

### Flow cytometry

Cell apoptosis and the expression of c-MET were examined by flow cytometry. Experiments included the following treatment groups: blank control group, radiation alone group, erlotinib alone group, and combined erlotinib and radiation group. The concentrations of erlotinib and the anti-c-MET monoclonal antibody were the same as those listed in the preceding section. All irradiated groups were given a dose of 6 Gy. Colony-forming cells from different treatment groups were collected, and the apoptotic rates and expression of c-MET were determined by flow cytometry. Apoptosis was measured experimentally as follows: first, cells were trypsinized, and 5 × 10^5^ cells were collected. After adding 1 ml of cold phosphate buffered saline (PBS), the cells were centrifuged at 1000 rpm for 10 min at 4°C. The cells were then washed with PBS, centrifuged twice under the above conditions, and resuspended in 200 μl of binding buffer. Ten microliters of Annexin-FITC was added to the cell suspension and mixed well. The cell mixture was incubated at room temperature in the dark for 15 min, and then an additional 300 μl of binding buffer was added. Finally, the cells were analyzed by flow cytometry after adding 5 μl of PI. To detect the expression of c-MET, the cells were incubated with the PE-coupled mouse anti-human c-MET monoclonal antibody, washed, incubated with the FITC-coupled rabbit anti-mouse antibody, washed, and then subjected to flow cytometry analysis [11,12].

### Western blotting

The expressions of c-MET, p-c-MET, PI3K, AKT, and p-AKT in the blank control group, the combined erlotinib and radiation group, and the combined erlotinib and radiation with anti-c-MET monoclonal antibody group were examined using Western blotting,. The concentrations of erlotinib and the anti-c-MET monoclonal antibody were the same as those described above. Cells were irradiated at a dose of 6 Gy. The experimental procedures were performed as follows: 14 days after treatment, the cells were trypsinized and collected. The total protein was extracted from the cells, and the protein concentration was determined by Coomassie brilliant blue staining. The proteins were separated by polyacrylamide gel electrophoresis and transferred onto polyvinylidene difluoride membranes. The membranes were then probed with primary antibodies, washed, incubated with horseradish peroxidase–conjugated secondary antibodies, and washed again. Finally, protein signals were visualized [13,14].

### Statistical analysis

Origin7.5 software was used to fit the cell survival curves. Data were presented as the mean ± standard deviation and were analyzed using SPSS17.0 software. The analysis of variance (ANOVA) tables were used to perform comparisons between multiple groups. P values less than 0.05 were considered statistically significant.

### Patient consent

The study was not related with patients.

## Results

### Apoptosis and colony formation under the combined treatment of erlotinib and 6 Gy of radiation

**Figure 1:**
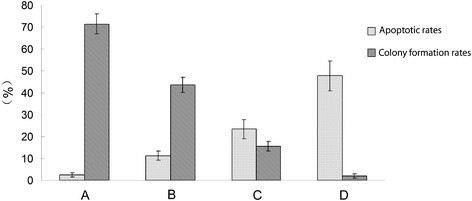
**Apoptosis and colony formation under the combined treatment of erlotinib(20 nM) and 6 Gy radiation. (A)** Control group: the apoptotic rate was 2.43 ± 1.03%; the colony formation rate was 71.45 ± 4.64%. **(B)** Erlotinib group: the apoptotic rate was 11.26 ± 2.14%; the colony formation rate was 43.56 ± 3.38%. **(C)** Radiation group: the apoptotic rate was 23.45 ± 4.35%; the colony formation rate was 15.6 ± 2.26%. **(D)** combined treatment with erlotinib and radiation group: the apoptotic rate was 47.68 ± 6.73%; the colony formation rate was 2.04 ± 1.02%.

Our results showed that the apoptotic rates of cells in the blank control group, erlotinib alone group, radiation alone group, and combined erlotinib and radiation group after 24 hours of treatment were 2.43 ± 1.03%, 11.26 ± 2.14%, 23.45 ± 4.35%, and 47.68 ± 6.73%, respectively. The apoptotic rate in the combined treatment group was significantly higher than those in the other groups (ANOVA analysis, P < 0.05). The number of colonies was counted after 14 days of treatment, and the colony-forming rates of cells in the blank control group, erlotinib alone group, radiation alone group, and combined erlotinib and radiation group were 71.45 ± 4.64%, 43.56 ± 3.38%, 15.6 ± 2.26%, and 2.04 ± 1.02%, respectively. Although the colony-forming rate in the combined treatment group was the lowest (Figure 1, ANOVA analysis, P < 0.05). These results showed that erlotinib exhibited a clear radiosensitizing effect. However, a portion of the tumor cells survived the combined treatment with erlotinib and radiation and eventually formed colonies.

### The expression of c-MET in colony-forming cells after the combined treatment with erlotinib and radiation

**Figure 2:**
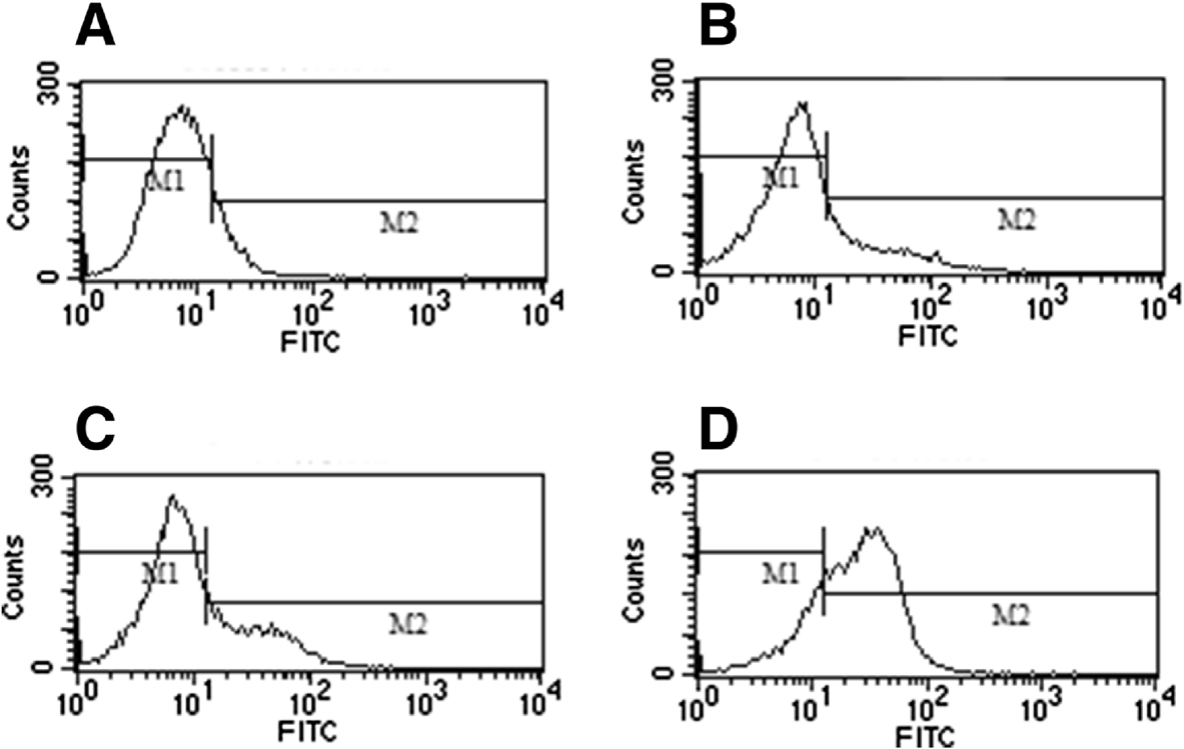
**The expression of c-MET in colony-forming cells after combined treatment with erlotinib (20 nM) and radiation (6Gy).** M1: the cell of c-MET non-expression. M2: the cell of c-MET expression. The expressions of c-MET (M2) were as follows: **(A)** Control group: 12.25 ± 2.17%. **(B)** Erlotinib group: 23.36 ± 3.16%. **(C)** Radiation group: 27.45 ± 3.79%. **(D)** combined treatment with erlotinib and radiation group: 75.35 ± 6.33%.

Cells from the blank control group, the erlotinib alone group, the radiation (6 Gy) alone group, and the combined erlotinib and radiation group after 14 days of treatment were collected, and the results of the flow cytometry analysis showed that the expression rates of c-MET in these groups were 12.25 ± 2.17%, 23.36 ± 3.16%, 27.45 ± 3.79%, and 75.35 ± 6.33%, respectively. Compared to other groups, the expression rate of c-MET in the combined treatment group significantly increased (Figure 2, ANOVA analysis, P < 0.05). These results showed that both radiation and erlotinib were contributing factors to the increased c-MET expression. Moreover, the combination of radiation and erlotinib had a synergistic effect on the upregulation of c-MET expression.

### The inhibition of c-MET activity further enhances the radiosensitizing effect of erlotinib

**Figure 3:**
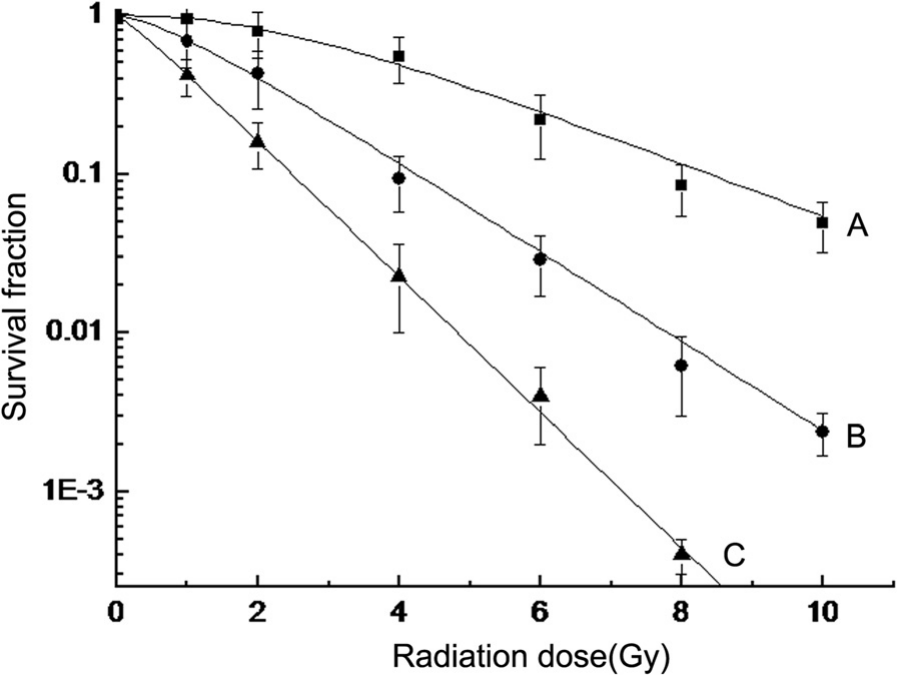
**Inhibition of c-MET activity further enhances the radiosensitizing effect of erlotinib.** Compared to the combined erlotinib(20NM) and radiation group, inhibition of the c-MET signaling pathway using a monoclonal antibody(10 nM) further decreased the survival fraction and enhanced the radiosensitizing effect (ANOVA analysis, P < 0.05) **(A)** Radiation group. **(B)** Combined erlotinib and radiation group. **(C)** Combined erlotinib and radiation with anti-c-MET monoclonal antibody treatment group.

The survival curves showed that erlotinib exhibited a clear radiosensitizing effect. Compared to the combined erlotinib and radiation group, the inhibition of the c-MET signaling pathway in these cells using a monoclonal antibody further decreased the SF and enhanced the radiosensitizing effect (ANOVA analysis, P < 0.05, Figure 3). The D_0_, N, and Dq values for the radiation alone group were 2.51, 2.93, and 1.17, respectively, and for the combined erlotinib and radiation group were 1.54, 1.62, and 0.32, respectively; the sensitizer enhancement ratio was 1.63. The D_0_, N, and Dq values for the combined erlotinib and radiation with anti-c-MET monoclonal antibody treatment group were 1.01, 1.17, and 0.07, respectively, and the sensitizer enhancement ratio was 2.49. Therefore, the elevated c-MET expression likely acted as a survival factor for the tumor cells treated with a combination of erlotinib and radiation, and the blockade of c-MET signaling significantly enhanced the tumor cytotoxicity effects.

### Changes in protein expression related to the c-MET-PI3K-AKT pathway in colony-forming cells from the combined erlotinib and radiation group before and after c-MET inhibition

**Figure 4:**
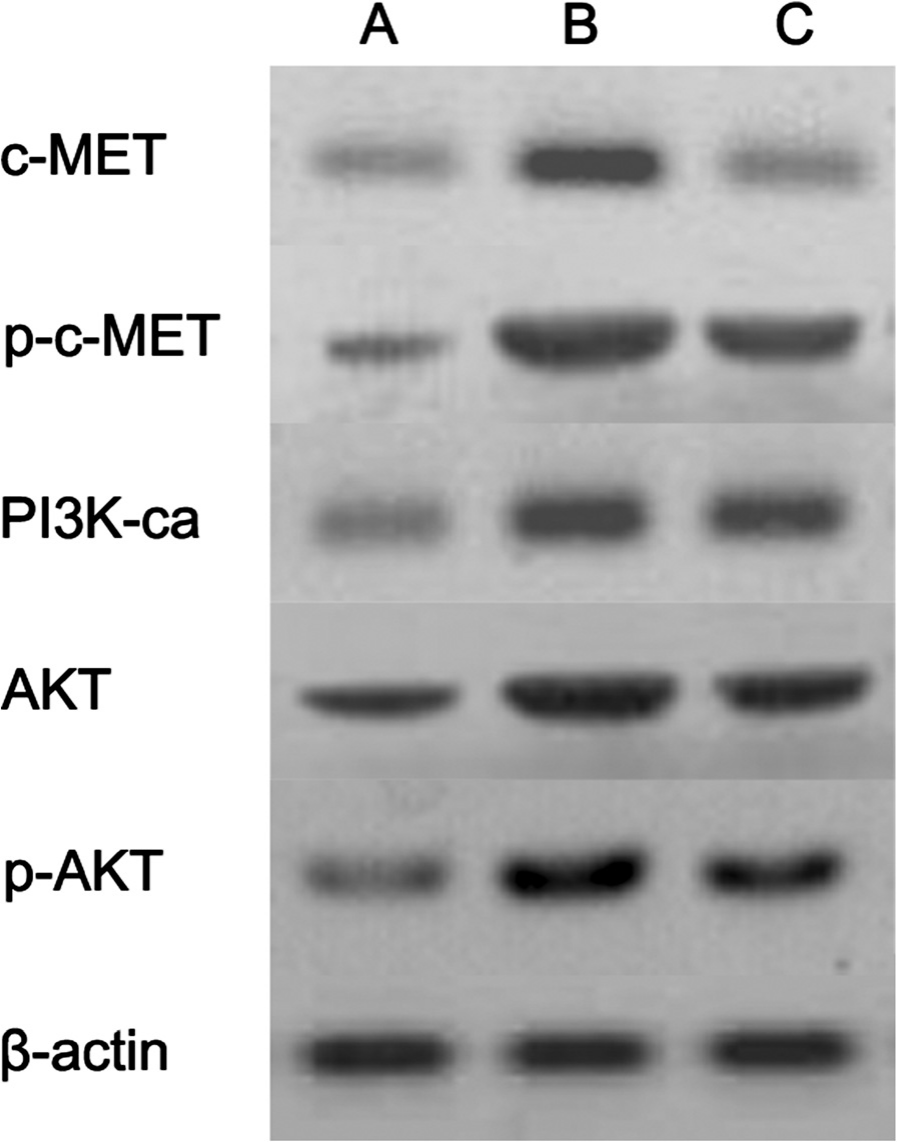
**Protein expression in colony-forming cells from the combined erlotinib and radiation group before and after c-MET inhibition.** The radiation dose was 6Gy. Erlotinib was used at a concentration of 20 nM. The anti-c-MET monoclonal antibody was applied at a concentration of 10 nM too. **(A)** Control group. **(B)** Combined erlotinib and radiation group. **(C)** Combined erlotinib and radiation with anti-c-MET monoclonal antibody treatment group.

To study the possible survival mechanism utilized by colony-forming cells, cells from the blank control group, the combined erlotinib and radiation group, and the combined erlotinib and radiation with anti-c-MET monoclonal antibody group were collected after 14 days of treatment, and the expression of c-MET, phosphorylated c-MET, PI3K, AKT, and phosphorylated AKT was examined. The results showed that the expression of these proteins significantly increased after 14 days of treatment. However, after treatment with the anti-c-MET monoclonal antibody, the expression of these proteins significantly decreased (Figure 4). Moreover, the changes in protein expression observed before and after inhibiting the c-MET-PI3K-AKT pathway using the anti-c-MET monoclonal antibody further indicated that the activation of the c-MET-PI3K-AKT pathway was an important mechanism for A973 cell survival and colony formation after the combined treatment with erlotinib and radiation. Therefore, blockade of this pathway may represent a novel approach for enhancing the radiosensitizing effect of erlotinib.

## Discussion

The results of the present study indicated that the activation of the c-MET-PI3K-AKT pathway served as an important survival mechanism for cells that received the combined treatment with erlotinib and radiation. However, blockade of the c-MET-PI3K-AKT pathway could further enhance the radiosensitizing effect of erlotinib.

Both erlotinib and radiation play important roles in activating the c-MET-PI3K-AKT pathway, which is one of the main causes of acquired resistance to erlotinib. After erlotinib treatment, the PI3K signaling pathway is significantly upregulated in tumor cells. Consequently, downstream pathways and nuclear gene expression are activated, resulting in tumor cell survival and proliferation [15,16]. In addition, many studies have shown that radiation stimulates the PI3K pathway [17-20], leading to radiation resistance in tumor cells. Under the combined action of erlotinib and radiation, the likelihood of c-MET-PI3K-AKT pathway activation significantly increases. Based on these findings, we selected c-MET-PI3K-AKT pathway to evaluate in our research. The mechanism by which the activated c-MET-PI3K-AKT pathway promotes tumor cell survival involves cross-talk between cell signaling pathways [21-24]. Normally, the EGFR/MAPK (mitogen-activated protein kinase) pathway is one of the major pathways driving tumor cell proliferation, whereas the activity of the PI3K pathway decreases due to the function of phosphatase and tensin homolog (PTEN). Upon erlotinib treatment, the MAPK pathway is inhibited and the expression of PTEN is downregulated, resulting in the decreased inhibition of the PI3K pathway [25,26]. Owing to the cross-communication between these pathways, the PI3K pathway is stimulated. Similarly, radiation further enhances the activation of the PI3K pathway. As a result, the activity of the PI3K pathway increases significantly, which maintains cell survival and proliferation [27,28].

The results of the present study support those of previous studies mentioned in the preceding paragraph, because in the present study erlotinib exhibited a clear radiosensitizing effect. However, many cells survived under the combined effect of radiation and erlotinib. The survival of such cells may be because both radiation and erlotinib can promote the expression of c-MET, which subsequently activates downstream pathways and affects their cytotoxic effects. Our finding that the inhibition of c-MET with a monoclonal antibody decreased the SF of tumor cells and downregulated the c-MET-PI3K-AKT pathway activity further confirmed the mechanism of tumor cell survival, and these results may provide a new approach to further enhance the cytotoxic effects of combined therapy with erlotinib and radiation in tumor cells (Figure 3).

Previous studies have mainly focused on the molecular mechanism of erlotinib-induced radiosensitization and cytotoxicity [2,3,29], and few have studied the survival mechanism of tumor cells given the combined treatment with erlotinib and radiation. Furthermore, studies exploring the connection between erlotinib-induced radiosensitization and the related signaling pathways are even rarer [30,31]. However, our present study sought to address these shortcomings, and we evaluated the c-MET-PI3K-AKT pathway to study protein expression changes following the combined treatment with radiation and erlotinib, as well as the regulatory effects of blocking of this pathway on erlotinib-induced radiosensitization. Furthermore, the current study preliminarily explored the survival mechanism of tumor cells receiving the combined treatment with erlotinib and radiation and provided constructive ideas for how to enhance the radiosensitizing effect of erlotinib.

## Current and future developments

In summary, this study performed constructive investigations of changes in the activity of the c-MET-PI3K-AKT pathway under the combined treatment with erlotinib and radiation and the regulatory effects of blocking the c-MET-PI3K-AKT pathway on the radiosensitizing effect of erlotinib. Our findings revealed an important mechanism for cell survival under the combined treatment with erlotinib and radiation and provided a new experimental basis for further enhancing the radiosensitizing effect of erlotinib. Although only *in vitro* experiments were performed in this study and many questions remained to be fully explored, we believe that with further in-depth basic research, erlotinib-induced radiosensitization will achieve superior efficacy and possess a broad clinical application.

## Abbreviations

EGFR: Epidermal growth factor receptor
MET: Mesenchymal-epithelial transition factor
PI3K: PI 3-kinases
FITC: Fluorescein isothiocyanate
